# Evaluation of Online Information in University Students: Development and Scaling of the Screening Instrument EVON

**DOI:** 10.3389/fpsyg.2020.562128

**Published:** 2020-12-16

**Authors:** Carolin Hahnel, Beate Eichmann, Frank Goldhammer

**Affiliations:** ^1^DIPF | Leibniz Institute for Research and Information in Education, Frankfurt, Germany; ^2^Centre for International Student Assessment (ZIB), Frankfurt, Germany

**Keywords:** evaluating online information, link selection, information relevance and credibility, university students, test development and validation

## Abstract

As Internet sources provide information of varying quality, it is an indispensable prerequisite skill to evaluate the relevance and credibility of online information. Based on the assumption that competent individuals can use different properties of information to assess its relevance and credibility, we developed the EVON (evaluation of online information), an interactive computer-based test for university students. The developed instrument consists of eight items that assess the skill to evaluate online information in six languages. Within a simulated search engine environment, students are requested to select the most relevant and credible link for a respective task. To evaluate the developed instrument, we conducted two studies: (1) a pre-study for quality assurance and observing the response process (cognitive interviews of *n* = 8 students) and (2) a main study aimed at investigating the psychometric properties of the EVON and its relation to other variables (*n* = 152 students). The results of the pre-study provided first evidence for a theoretically sound test construction with regard to students’ item processing behavior. The results of the main study showed acceptable psychometric outcomes for a standardized screening instrument with a small number of items. The item design criteria affected the item difficulty as intended, and students’ choice to visit a website had an impact on their task success. Furthermore, the probability of task success was positively predicted by general cognitive performance and reading skill. Although the results uncovered a few weaknesses (e.g., a lack of difficult items), and the efforts of validating the interpretation of EVON outcomes still need to be continued, the overall results speak in favor of a successful test construction and provide first indication that the EVON assesses students’ skill in evaluating online information in search engine environments.

## Introduction

Information literacy and related competencies have become essential in the digital era, as they refer to skills and knowledge that students need in order to act effectively, confidently, and successfully in dynamic and interconnected information environments. However, there is an urgent need to improve students’ information literacy beyond simply making necessary tools and resources available. For example, according to the international large-scale assessment ICILS (Fraillon et al., 2020), only a small percentage of the participating school students were able to critically evaluate and use information when searching online (see also Breakstone et al., 2019). University students, who are expected to possess a certain level of competence (Association of College and Research Libraries, 2000), are no exception to this phenomenon. Studies indicate difficulties in identifying information and information sources that are reliable and trustworthy (e.g., Walraven et al., 2008; Maurer et al., 2017), but there are efforts to support students in developing their information literacy (e.g., Peter et al., 2017; McGrew et al., 2019). One recent European example is the multilingual Massive Open Online Course (MOOC) of the Erasmus+ project Information Literacy Online (ILO; Mandl et al., 2018)^1^. This MOOC provides students with open learning materials, quizzes, and achievement tests for self-assessment purposes. The EVON is one of those achievement tests, with the aim of giving students a first impression of their performance in evaluating the relevance and credibility of online information from search engine results—a central component skill of information literacy. In this article, we report on the test development and first efforts to validate the interpretation of its test score (i.e., construct interpretation).

## Evaluating and Selecting Online Information

Processing, evaluating, and deciding on the use of information during a web search is a complex phenomenon. Accordingly, there are various interdisciplinary approaches in research, often focusing on selected aspects. In this section, we give a short introduction to different conceptualizations, theories, and related empirical observations. We start with a formal description of the web search process and elaborate on when evaluations are triggered in this process, what purposes they serve and why their depth will vary depending on the context (see section “Web Search as a Decision-Making Problem”). We then go into detail about how individuals determine the relevance of information for a particular task (see section “Determination of Relevance”) and how they make credibility assessments of information and information sources (see section “Determination of Credibility”). We conclude the introduction with a short overview of previous assessment approaches that capture how individuals assess the relevance and credibility of information (see section “Assessment Approaches”).

### Web Search as a Decision-Making Problem

Search engines usually provide web users with large amounts of information that can relate to a topic of interest in many different ways (see e.g., Bendersky et al., 2012). In procedural descriptions of the web search process, such as the IPS model (information problem solving; Brand-Gruwel et al., 2005, 2009), it is distinguished that web search requires individuals to (1) identify their information needs; (2) specify their search strategy and select links on a search engine result page (SERP) based on initial judgments; (3) scan the information on the websites visited to get an idea of whether it could be useful; (4) deeply process the information identified as useful in the previous step and integrate it with previously found information and prior knowledge; and (5) compare and integrate all collected information to form some kind of response. Steps (2) to (4) require web users to evaluate information in order to decide which information object should be selected from multiple alternatives and considered as part of a response.

The assessment of relevance and credibility is considered an iterative process in which a person makes a series of judgments about the available information (Hilligoss and Rieh, 2008). The scientific literature mainly distinguishes between two types of judgment, which serve different purposes: Predictive judgments are made before accessing the object of evaluation (e.g., a website); evaluative judgments are made when confronted with the object of evaluation (Rieh and Danielson, 2007). Predictive judgments are used to anticipate the value of information for a task and to decide whether or not to follow a SERP link or consider a particular website. A web user’s perception of the value of information (“information scent”) is obtained by cues in the immediate task environment (“proximal cues”; e.g., Sundar et al., 2007). Such cues can manifest themselves in many ways, for example, semantically (e.g., keywords from the search query; Rouet et al., 2011) or by describing structural, message-related and sponsor-based features of information (e.g., website layout, topicality, or source reputation; Metzger and Flanagin, 2013). They are often only examined for the first few entries of a SERP, which indicates an implicit trust in the optimization of search engine algorithms (e.g., Pan et al., 2007; Kiili et al., 2008; Walraven et al., 2008; Kammerer and Gerjets, 2014). Failure to find “valuable” information is more likely to prompt web users to modify their search query rather than to continue examining other SERP entries (e.g., Huang and Efthimiadis, 2009; Hollink et al., 2012). Accordingly, predictive judgments represent in some way a “bouncer” in deciding whether information should be processed at all, with the accessibility and interpretability of cues being crucial to this decision. This also means that web users may omit important information or turn to less suitable information if their predictive judgments are inadequate (see Kiili et al., 2008). Evaluative judgments, in contrast, serve to determine whether and how identified information is suitable for solving the information problem. If individuals come to the conclusion that the information is of value for providing a sufficient outcome (Pirolli and Card, 1999), they will process this information in further detail and integrate it as part of fulfilling their search task. If not, the website is likely to be discarded (e.g., Salmerón et al., 2017).

The depth and level of detail of evaluations made will depend on the way in which web users process the identified cues. Dual-processing theories (e.g., Wirth et al., 2007) distinguish between systematic processing, which involves a relatively analytical, thorough, and comprehensive examination of information versus heuristic processing, which is fast and automatic and does not consume too much processing resources (e.g., time and attention). They suggest that online information is not fully processed, with the result that individuals use cognitive “shortcuts” based on the cues considered (Gigerenzer and Gaissmaier, 2011). Similar predictions are made based on information foraging theory (Pirolli and Card, 1999) that postulates that web users search in a way to maximize their gain of valuable information while keeping their effort as low as possible. Depending on the context, however, heuristics can be inadequate, leading to erroneous assessments (e.g., Rouet et al., 2011; Metzger and Flanagin, 2013).

Web users will primarily select information based on its relevance to the task at hand (see Rouet, 2006; Kiili et al., 2008), although a concurrent critical evaluation of source characteristics of information is indispensable as it can help individuals to avoid misinformation and overcome misconceptions (overview in Braasch and Graesser, 2020). A source of information might be recognized as credible, but is unlikely to be considered further if it does not provide any indication of relevance. Accordingly, relevance assessments traditionally are important criteria for assessing the credibility of information (Rieh and Danielson, 2007). Nevertheless, in order to understand the mechanisms of individuals’ assessment of relevance and credibility, it is useful to consider both aspects in their own right.

### Determination of Relevance

Relevance concerns the extent to which information matches the needs given the specifications of a task (McCrudden et al., 2005). Accordingly, the degree to which information segments are evaluated as relevant will mainly depend on a web user’s search goal. To determine relevance, web users will rely on the use of surface cues and deep semantic cues that require decoding and comprehension. They can benefit from both types, although an overreliance on superficial cues can result in neglecting important aspects. There is evidence that adolescents show increasing skill in recognizing deep semantic cues over time (Rouet et al., 2011). Compared to older students, early secondary school students tended to rely more on surface cues (e.g., keywords that are written in upper cases), indicating that younger students experience more difficulties in balancing the use of surface and deep cues when selecting website titles (ibid.). Keil and Kominsky (2013) came to a similar conclusion studying how 11-year-olds to over 18-year-old students increasingly include discipline-related cues in their evaluation of search results. The recognition of deep conceptual relationships between a search task and a search result that are not entirely obvious (i.e., due to the absence of lexical similarity on the surface) increased over high school years and received a level in grade 10 that was comparable to adult-like performance. Although an overreliance on surface cues seems to decrease over time, it remains crucial that web users do not falsely determine relevance from an uncritical use of surface cues.

Besides prior knowledge (e.g., Hölscher and Strube, 2000), other important factors that influence how web users determine the relevance of information clearly concern information processing skills (or conditional skills in the IPS framework; Brand-Gruwel et al., 2009), such as reading. Reading skills support web users in identifying and locating relevant information, for example, by enabling them to extract main ideas from text (Hahnel et al., 2016). Highly skilled readers also seem to be in a better position to identify deep semantic cues and make use of them to efficiently discard irrelevant information (Hahnel et al., 2018). However, this does not necessarily mean that skilled readers are also skilled searchers. Salmerón et al. (2017) found that if skilled readers fell for irrelevant sections of a digital text, they were at a greater disadvantage than less skilled readers, indicating that skilled readers do not automatically recognize deep semantic cues correctly or sufficiently process them.

### Determination of Credibility

Traditional “gatekeepers” such as editors, reviewers, and publishers are often not available to ensure the integrity of online information (Flanagin and Metzger, 2007; Rieh and Danielson, 2007). Accordingly, the recognition of credibility aspects of information has become increasingly necessary, in particular when information is presented in a way that resembles editorial content but is paid for by an advertiser (sponsored content as part of native advertising; see Amazeen and Muddiman, 2018). This is a difficult task for students, even when the advertisements are explicitly marked (Wineburg et al., 2018). Students rarely spontaneously evaluate credibility aspects of information obtained (for an overview, see Bråten et al., 2018), and although they tend to select information from seemingly credible sources, students lower their evaluation standards if they do not have access to better information sources (Kiili et al., 2008).

According to feature or checklist approaches (Flanagin and Metzger, 2007; Metzger, 2007; see also Chinn and Rinehart, 2016; van Zyl et al., 2020), web users’ perception of credibility will depend on their judgments referring to structural (e.g., design features and website complexity), message-based (e.g., accuracy and writing style), and sponsor-based features (e.g., personal experience with the sponsor). The weight given to each feature may vary depending on the genre of website or other circumstances (e.g., websites from news organizations are generally rated more credible than personal websites; Flanagin and Metzger, 2007). It is noteworthy that we distinguish between semantic cues and structural, message-based and sponsor-based features, although there is a strong conceptual overlap in the properties addressed. This is done with the purpose of distinguishing whether a cue or feature is primarily used to determine relevance or credibility. For example, recognizing the intention of a text will inform both the assessment of relevance and credibility, but might be evaluated with an emphasis either on whether the content can contribute to solving the information problem or whether the text has secondary motives.

The recognition and use of specific features are assumed to trigger heuristics to aid the assessment of credibility (Metzger and Flanagin, 2013). Accordingly, participants, interviewed in focus groups, showed to employ a wide variety of cognitive heuristics, which Metzger et al. (2010) classified as rooted in social confirmation (e.g., reputation heuristics, such as the rule of thumb that URLs of .org domains are credible) or rooted in expectancies within specific contexts (e.g., persuasive intent heuristics, such as the presence of advertisements as negative credibility indicators). Although such heuristics are often helpful, they can still lead to biased assessments, for example, when information is dismissed as not credible only because of discrepancies with one’s own beliefs or those of peers and vice versa (see Braasch and Graesser, 2020).

Checklist approaches imply that information credibility is determined by whether or not the information and its source show certain characteristics. It should be noted that Chinn and Rinehart (2016) argue that such characteristics are only valid if they actually correspond to the use of reliable epistemic processes to produce knowledge claims. That means, for example, that a news website should be considered credible not because it is operated by a news agency, but because its journalists produce knowledge claims that are accurate and plausible in their argumentation, which rely on processes of thorough search, evaluation, and synthesis of evidence to produce them. Recent considerations support this view arguing that core components of critical thinking (e.g., evaluating whether a claim is validated by examining the argument surrounding it) can enrich checklist approaches and should be considered to foster students’ credibility assessment (van Zyl et al., 2020; see also Stadtler and Bromme, 2014, on strategies to reconcile conflicts about competing scientific claims). Nevertheless, provided that they are closely related to such epistemic processes, structural, message-based, and sponsor-based features are useful markers that present web users with comparatively simple and straightforward ways to assess the credibility of information.

### Assessment Approaches

Many instruments claim to assess information literacy, which emphasizes the importance of this construct in research and society. In an attempt to structure the field, Walsh (2009) reviewed 91 scientific articles, summarizing several approaches to assess information literacy. He identified in total nine different methodologies (e.g., essays, observations, portfolios, “self-assessments” in the sense of self-report). Most prominently were multiple-choice questionnaires and quizzes, but Walsh remarks that the respective studies have often not been thorough in their efforts to investigate the reliability and validity aspects of their instrument (see also Rosman et al., 2016, for a discussion of different test formats).

Recent approaches are increasingly focusing not only on declarative knowledge aspects of students’ information literacy, but also on procedural knowledge and actual behavior. We briefly highlight some instruments of information literacy that we think have a convincing approach. For example, Leichner et al. (2014) suggested a taxonomy to create information search tasks that request students to find a scientific article about a subject. After each task, the students are asked several questions about their task processing, which serves as the basis of scoring students’ procedure. Rosman et al. (2016) proposed a less resource-consuming vignette-based approach. They constructed a test of 28 situational judgment tasks that provided students with a scenario description and several possible procedures to solve the scenario and requested them to rate each procedure according to its usefulness. Also worth mentioning is the serious game of Steinrücke et al. (2020). They measured information literacy by classifying the in-game behavior of individuals playing a crisis situation manager game. However, their validation approach strongly relied on a self-report, not an independent performance measure.

Especially students’ evaluation of information from search engines is often examined based on their performance in open search tasks of varying complexity (e.g., fact-finding vs. research-oriented tasks, closed-ended vs. open-ended tasks; e.g., Wirth et al., 2007; Kiili et al., 2008; Brand-Gruwel et al., 2009; Bilal and Gwizdka, 2018; Pardi et al., 2020). The assessment, scoring, and evaluation of performance are usually recorded by an additional tracking application, such as screen recording or a proxy server that retrieves search engine data in the background. Although such task setups can provide substantial information about the evaluation skills of individuals, they are often not standardized or lack controlled and comparable conditions. Therefore, a number of researchers have moved toward the development of search tasks in mock environments. That means they have created search engine results and/or websites that were identical for all participants or groups of participants to ensure comparability (e.g., Rouet et al., 2011; Keil and Kominsky, 2013; Metzger and Flanagin, 2013; Kammerer and Gerjets, 2014). Such simulation-based approaches are also often used to assess constructs that are closely related to information literacy, such as individuals’ skills in dealing with information and communication technologies (e.g., ICILS, Fraillon et al., 2019; for an overview see Siddiq et al., 2016), problem-solving in technology-rich environments (e.g., Goldhammer et al., 2020), digital reading (OECD, 2011), or skills in online research and comprehension (ORCA; e.g., Leu et al., 2014).

A simulation-based approach was also implemented by Keßel (2017) to test the evaluation skill of adolescents (see also Hahnel et al., 2018). She developed 24 items that simulated search results and Internet forums in which students were requested to identify and select the most credible entry for the respective search task. Eight of these items presented students with a page of search results (SERP) related to topics on health, crafts, sports, and education. The items were interactive, as students are allowed to access a website through the links, providing them with detailed information. A correct answer was defined by the search result (i.e., the target) with the highest number of features that identified it as credible. The items varied according to the attractiveness of non-target search results (low vs. high attractiveness) and the congruence of features indicating the credibility of the source underlying the search results (congruence vs. incongruence). Keßel defined these criteria based on the number of features that indicate the credibility of the SERP results (attractiveness) and based on whether the information of a SERP result and its corresponding website signal a similar degree of credibility (congruence). Inspired by her instrument, we developed the EVON (evaluation of online information) to assess the evaluation skill of students in higher education.

## Framework and Test Development

Based on the theoretical background, we define the skill to evaluate online information as the cognitive skill to recognize and make use of semantic cues and structural, message-based, and sponsor-based features in order to evaluate the relevance and credibility of information in search engine environments (after Keßel, 2017). We assume that students who engage in web search first scan a SERP and generate a series of predictive judgments to preselect websites for close examination (Rieh and Danielson, 2007; Brand-Gruwel et al., 2009). When a website is accessed, we assume that students make evaluative judgments to determine the extent to which the website contributes to the completion of their search task. If a decision has to be made between several positively evaluated alternatives, the identified relevance and credibility aspects are compared and weighed against each other. Accordingly, a student competent in evaluating online information is able to select websites suited for a specific task based on informed conclusions about the relevance and credibility of information. A test that claims to assess how students evaluate online information should therefore take this process into account and provide students with opportunities to judge different features of links and websites of varying relevance and credibility. In the following, we describe the development of the interactive computer-based instrument EVON, which aims to provide students who wish to improve their information literacy (Mandl et al., 2018) with a screening of their evaluation skills.

### Guidelines for Item Design

The EVON was designed to request students to select the most relevant and credible link in a simulated search engine environment for a respective task. Accordingly, we have adopted the basic task structure of Keßel’s (2017) items simulating a SERP and websites. However, we have decided to emphasize the role of relevance assessment because it is likely that information in web search contexts will not be further processed if it is not found to be related to a task at hand. Although checklist approaches consider relevance as part of the credibility assessment (especially with regard to message-based features), we intended to acknowledge in particular situations where websites can be credible but may be not relevant and vice versa.

The new items were designed to present a target that is the optimal solution in terms of both relevance and credibility of information. Competing non-targets were characterized by flaws and shortcomings compared to the target. In the revision process, we made sure that the provided cues and features were consistent with the expected epistemic processes (e.g., if a website was authored by an expert, the knowledge claim would be accurate; see Chinn and Rinehart, 2016). Table 1 summarizes the combinations of the two main design criteria, attractiveness and congruence. However, we have broadened the definition of Keßel’s (2017) design criteria to explicitly consider relevance aspects and implications for the expected item solution process. For each of the four resulting types, two tasks were developed that presented either three or five information sources on a SERP.

**TABLE 1:** Guidelines for item design.

**Item type**	**Guiding characteristics**	**Description**	**Expectation for the solution process**
1	Low attractiveness of non-targets. Congruence between link and website	The target link already stands out from the non-target links in terms of features signaling relevance and credibility	Navigation is not necessary, as predictive judgments are sufficient, but can consolidate a decision
2	High attractiveness of non-targets. Congruence between links and websites	The target differs only marginally from non-targets in features signaling its relevance and credibility	Individuals need to judge and consider several aspects of information from both link and website to identify the best option
3	High attractiveness of non-targets. Incongruence between target link and website	The target link differs only marginally from non-target links in features signaling its relevance and credibility, but its website stands out compared to non-targets	Individuals can identify the target as the best option by inspecting its website
4	High attractiveness of non-targets. Incongruence between non-target links and websites	The target link differs only marginally from non-target links in features signaling its relevance and credibility, but the non-target websites violate the expectations generated by their links	Individuals can exclude non-targets by inspecting their websites

The attractiveness criterion addresses the extent to which non-target SERP links display cues that affect their perceived information value. Non-target links of low attractiveness are only superficially related with a search task (item type 1; e.g., when searching for a solution to an email attachment problem, the results not only present a link addressing the problem, but also a link about dangerous attachments in phishing emails). As in these conditions students can potentially identify the target based on predictive judgments, these tasks are supposed to be the easiest tasks. In contrast, highly attractive non-target links signal an information value similar to the target link, which means that predictive judgments cannot be used exclusively to identify the best source of information (type 2; e.g., when searching for information about diving equipment for beginners, the results present a link about basic equipment and links about special equipment). Accordingly, a high non-target attractiveness is expected to increase the item difficulty.

The congruence criterion addresses the extent to which SERP links can raise expectations that may be violated by the information on the website. Because of the extended scope compared to the definition of Keßel (2017), we considered that with regard to authentic web search situations, this criterion is only meaningful for non-targets that are as attractive as the target (i.e., the condition of high attractiveness). With respect to the incongruence condition, the most significant change that we made was to indicate the object and the direction of incongruence. That means we distinguished between situations in which the target link (type 3) or the non-target links (type 4) violate the expectations formed by predictive judgments. In type 3 items, the SERP presents a list of moderately useful-looking links (e.g., when searching for remedies against a cold, the SERP lists websites from a news agency, a pharmaceutical journal, or a discussion forum), with the target being clearly identifiable as suitable by the information on its website. In type 4 items, all links on the SERP indicate to provide useful information, but when visiting the non-targets, it becomes evident that their websites are less appropriate (e.g., they indicate primary commercial intentions or address a different audience). As students may need to reconsider their initial assessment of relevance and credibility after new (incongruent) information is discovered, the tasks of the incongruent conditions are supposed to be difficult, but visits to websites can facilitate the evaluation, as more information becomes available to make an informed decision.

**FIGURE 1:**
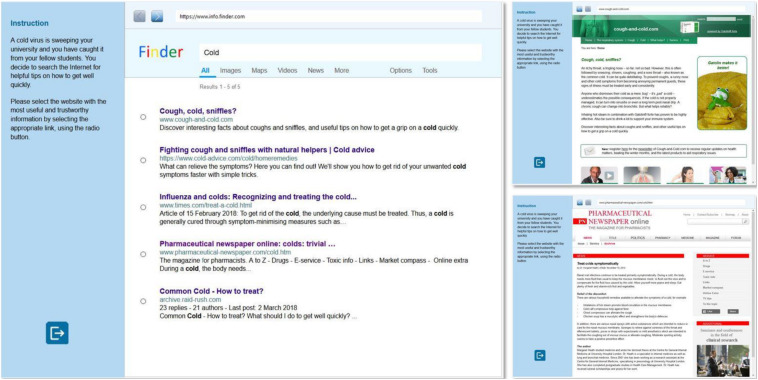
Screenshot of an EVON item with two exemplary websites.

An overview of all developed items is presented in Table 2 (with detailed information about the respective item type in Table 1). An example item is displayed in Figure 1. The item “Recovering from a cold” instructs students to search for useful and trustworthy information to treat a common cold. This item belongs to item type 3 (i.e., high attractiveness of non-targets, incongruence between target link and website). According to the high attractiveness condition, the search results on the SERP were created to appear equally suited to solve the underlying information problem (“get a grip on a cold quickly,” “get rid of your unwanted cold,” “What should I do to get well quickly,” etc.). A SERP of low attractiveness would require non-target search results to be only superficially related to the search task (e.g., with regard to the word “cold,” a website could refer to chronic obstructive lung diseases or a rock band). According to the target-incongruence criterion, the target website is supposed to stand out in terms of relevance and credibility. In case of the example item, the target link (“Pharmaceutical newspaper”) suggests that the website is directed to a professional audience, but when inspecting the website (and eventually comparing it to the other websites), it becomes clear that its information is suitable to solve the search task, information about the author and publisher is clearly stated, and it can be expected that the author and publisher have authority in the respective field. For comparison, in the case of congruence, the link would actually lead to a website with highly specific pharmaceutical information.

**TABLE 2:** EVON item overview.

**Item**	**Description**	**Item type**	**No. links**
1	Restoring the charging capacity of a laptop battery	1	3
2	Recovering from a cold	3	5
3	Writing a scientific paper	4	5
4	Repairing a broken bicycle chain	3	3
5	Finding out about basic equipment required for diving	2	5
6	Preparing for a stress-free examination period	4	3
7	Resolving the blocking of an email attachment	1	5
8	Financing a semester abroad	2	3
			

### The Developed Test

The developed items of the EVON cover different topics that were chosen in consultation with representatives of the target population to ensure that the topics are relevant and authentic (Table 2). Nevertheless, we aimed at constructing the test in a way that students had as little advantage as possible due to their prior knowledge. Accordingly, the contents are fictitious, with existing websites having served as loose templates. Mainly due to copyrights, we have also refrained from using real brand and organization names.

The EVON is a power test in which students are asked to perform at their best (see Klehe and Anderson, 2007). We aimed for a setting that was as authentic and unobtrusive as possible, but the purpose of the assessment is not masked in any way. The item instructions explicitly request students to select a link for a respective task with regard to relevance and credibility aspects (“[…] select the website with the most useful and trustworthy information […]”). Students’ performance is scored dichotomously to whether they selected the target or a non-target. During the test-taking process, mouse-click data with timestamps are collected in log files. An interactive tutorial introduces students to the environment and all available functionalities. We recommend a total test time of 18 min to complete the EVON assessment. The EVON was implemented with the software CBA ItemBuilder^2^ and is available in six different languages (German, English, Spanish, Catalan, Croatian, and Slovenian). The corresponding author can be requested for test uses and modifications.

### Examining the Intended Test Score Interpretation

Based on students’ information selection, the EVON claims to assess their skill to evaluate the relevance and credibility of online information in search engine environments. A first step to support this claim was taken with the theory-based design of the interactive and authentic task environment. To further ensure the quality of the assessment and to validate the intended interpretation of the EVON score, we conducted a pre-study during the phase of item development and a main study after the EVON item set was finalized. The overarching goal of these studies was to collect validity evidence from different sources that provide information on the perception of item content, response processes, the internal structure of the EVON, and the nomological network of its score, allowing to evaluate arguments for and against the intended interpretation of the EVON score (see American Educational Research Association et al., 2014).

With regard to the test construction, we investigated whether the items are suitable to elicit and observe information selection based on students’ assessment of relevance and credibility (pre-study). After finalizing the test development, we investigated the internal structure of the EVON and effects of the item design criteria on the item difficulty by means of a larger student sample (main study). To investigate evidence referring to the nomological network of the EVON score, the network of relations to construct-related variables was also examined (main study). We focused on the relationship of the EVON score to students’ general cognitive performance and basic reading skills, taking into account their self-reported prior knowledge of the EVON topics.

## Pre-study

### Aim of the Study

Cognitive interviews were carried out to observe the course of students’ processing of the constructed items. The objectives were twofold: First, the study served to ensure the comprehensibility of item content and the usability of the test environment. Second, it was investigated whether the presented semantic, structural, message-based, and sponsor-based cues were identified and used to assess the relevance and credibility of information. Adjustments were made in response to participants’ feedback on incomprehensibility and misconceptions (e.g., clarifying instructions, modifying link and website information to provide more or less relevance and credibility related cues).

### Method

We collected the data of eight students (five females; mean_age_ = 25.6 years; seven enrolled in a master’s program). The test sessions were organized individually and lasted for 1.5–2 h, depending on the participants’ speed. An interviewer welcomed and instructed the participants and monitored the session. After giving their written and informed consent, the participants were instructed to think aloud while working on the German version of the EVON. Camtasia Studio 6 was used to synchronously record participants’ voice and processing behavior (via screen capture). To familiarize participants with the think-aloud procedure, each session started with a warm-up task. If the participants stopped verbalizing their thoughts, the interviewer reminded them to keep talking (see van Someren et al., 1994). During the assessment, the interviewer took notes about a participant’s behavior (e.g., which link attracted the participant’s attention first, which link was ignored, or which websites were clicked but left quickly). After completing the EVON, the interviewer asked the participants questions about the appropriateness of the tutorial, the clarity of content and instructions, the authenticity of the simulated web environment, and any specificities identified during the session (e.g., why was a particular link ignored). The interviewer also asked the participants for an assessment of their prior knowledge of the EVON content, as well as demographic information (age, gender, study program, and semester). Afterward, the test session was completed, and participants could choose to receive course credit or a monetary compensation for their participation. The resulting screen-capture videos with the participants’ verbalized thoughts and their answers during the interview were transcribed. The transcripts and the interviewer’s notes were analyzed to determine if the items were processed as intended.

### Results and Discussion

The simulated web environment was generally perceived as authentic and natural, with only two remarks indicating astonishment that someone was looking for remedies for a common cold (remark by Charlotte^3^) or that only three results were returned from the search engine (Fiona). Overall, the responses and comments of participants suggested that they processed the EVON items as intended. During the processing of the EVON items, they commented on specific semantic, structural, message-based and sponsor-based properties of the EVON stimuli indicating that they recognized and interpreted these cues to determine the relevance and credibility of links and websites. In addition, they explicitly reported on their use of these cues during the interviews. Below, we illustrate our findings with selected interview snippets from the item “Recovering from a cold” (Figure 1).

Examples that indicate the use of surface and deep semantic cues for assessing information relevance are presented in Table 3. The participants demonstrated to integrate surface cues in their judgments by mentioning keywords in the SERP link (Alexander and Emily) or scanning the website (David and Fiona). Alexander’s and Emily’s comments are examples of predictive judgments that are generated to decide whether to visit or dismiss a particular website. In contrast, David’s and Fiona’s comments rather reflect evaluations to decide whether a website is worth reading thoroughly. David’s comment even incorporates the use of a message-based feature that backs up his decision with an initial credibility judgment of the website (“if a doctor even writes that”). The examples for the use of deep semantic cues suggest that the participants reflected deeply on how the encountered information contributes to solve the associated search task by evaluating it in light of their personal experiences and world knowledge (Alexander and Bianca) or in terms of whether the provided information meets the requirements of the search task (David and Henry).

**TABLE 3:** Indications for the use of semantic cues to determine information relevance.

**Cue**	**Example quotes**	
Surface	Alexander:	[Inspects link 1] “So… Cough, cold and sniffles [mumbles]. Okay, that sounds pretty good.”
	David:	[Scans website 4] “Hmm, treat symptomatically. OK, I’ll have a look. OK, it’s probably a newspaper… so if a doctor […] even writes that, then I would have a closer look.”
	Emily	[Inspects link 2] “[reads ‘Fight coughs and colds with natural helpers… relieve symptoms’] Well that would be something, it’s all about getting me healthy again quickly.”
	Fiona:	[Scans website 3] “Help with cold… bacterial infections… antibiotics may be necessary… allergic rhinitis… allergies… active ingredient… [pause]. Okay doesn’t quite seem to be it.”
Deep	Alexander:	[Website 2] “There are even some… Exactly, there are also recipes with which I can make myself something to drink or eat. And I know that with ginger, lemon juice, honey, yeah that should probably help.”
	Bianca:	[Website 2] “Is it important to drink a lot […] I find that good, because the doctor, when I am sick, always tells me: Drink, drink, drink. So this is also what the doctor advises me.”
	David:	[Website 3] “OK I don’t have a… yeah I don’t have an allergy. Well, that’s not very helpful.”
	Henry:	[After having visited websites 1 and 2] “So here the [link 1] was just an introduction, so I couldn’t really know if that helps what is offered there. Here [link 2] was at least directly something visible.”
		

Table 4 shows examples of how the participants referred to structural, message-based, and sponsor-based features of websites to infer on information credibility. Structural features mentioned referred to the presence of pictures (Bianca), the general layout of websites (Charlotte), or typesetting (Emily). Multiple features are sometimes blended and get integrated or weighted against each other, as the comments of Bianca and Emily demonstrate. In their comments, they refer to both structural features (“that medicine up there,” “such a small font”) as well as message-based (“relatively clear, actually explains”) and sponsor-based features (“looks like a commercial”). The comments classified as referring to message-based features show that the participants took different aspects into account when judging the message that a website intends to convey. They elaborated on the author’s background (Alexander), evaluated information in terms of its currency and the comprehensibility of information provided (Bianca), or considered whether information was legitimated by trusted authorities (Fiona; also Bianca’s comment in Table 3). With regard to sponsor-based cues, it might have been suspected that students would rather base their judgments primarily on structural and message-based features due to the lack of real brand and organization names (Flanagin and Metzger, 2007). However, sponsor-based cues were identified and taken into account, as shown by references to publishing organizations (Alexander and Giselle) or recognized expertise (Bianca).

**TABLE 4:** Indications for the use of features to determine information credibility.

**Feature**	**Example quotes**	
Structural	Bianca:	[Website 1] “That looks like a commercial to me with that medicine up there. […] I find it funny with the frog on the side [pause], but ‘Gatolin makes it better’… well that uh puts me off.”
	Charlotte:	[Website 2] “Well, the second page looks a bit trashy from a layout point of view, so not so reliable.”
	Emily:	[Website 4] “Yes, I find that quite… well, somehow it’s not so vivid, because there’s such a small font and all, but hmm. Well, [it is] relatively clear, actually explains what you can do anyway, but don’t find the page so likeable actually.”
Message-based	Alexander:	[Website 4] “Uh especially in the field of medicine there are many who simply tell something that doesn’t have to be true. […] it is good to know that at least a doctor wrote it and not just anyone.”
	Bianca:	[Website 3] “The source, ‘Internal differential diagnosis,’ OK. But the source is pretty old, from 1999!”
		[Inspects link 4] “Pharmaceutical newspaper, hmm magazine for pharmacists, okay I think that is… if it’s for pharmacists, it will probably be too complicated for me.”
	Fiona:	[Website 2] “Ginger tea usually always works well, says my mum.”
Sponsor-based	Alexander:	[Website 4] “So I would say, since really, uhm, the publisher is named, and ah it’s a serious publisher, I would prefer this source.”
	Bianca:	[Scans website 1] “Ok, with the expert interview I automatically think that this site works with experts and therefore is qualitative.”
	Giselle:	[Visited only website 4, retrospective interview] “That sounded trustworthy. Not because the others weren’t any good, […] I just had no reason to keep on searching […] it was published in a newspaper and, I don’t know, sounded better than [link 5].”

The overall impression gained from the participants’ comments is that they made use of several cues to infer both the relevance and credibility of the information provided and that they combined different heuristic strategies to process the EVON items, which is consistent with the assumptions of the test construction (e.g., Rouet, 2006; Brand-Gruwel et al., 2009; Metzger and Flanagin, 2013). In this respect, the results of the cognitive interviews provide first empirical evidence based on the item contents and the response processes observed, supporting the intended interpretation of the EVON score.

It should be noted that, in terms of performance, the participants showed high rates of correct responses (success rates per item between 50 and 88%). Accordingly, the test was rather easy. However, this might be due to the setup of cognitive interviews. As participants were asked to verbalize their thoughts and comment on the material as part of improving the items, they might have adopted a higher desired level of understanding the provided information and engaged in strategic rather than automatic processes of reading (standards of coherence; van den Broek et al., 2011). Accordingly, they might have reflected upon the links and websites more thoroughly than they would have done otherwise.

## Main Study

### Aim of the Study and Hypotheses

With the overarching objective of validating the interpretation of the EVON score, an online assessment was conducted to investigate the psychometric properties of the EVON and to test hypotheses relating to the design of its items and nomological network. With regard to the psychometric properties, it was expected that the EVON items contribute to the assessment of a unidimensional skill that is part of the broader construct of (online) information literacy. Support for the assumption of unidimensionality would allow for the differentiation of different skill levels in evaluating online information.

With regard to the item design (Table 1), we expected to find differences in item difficulty related to the item type and to whether or not students visited target or non-target websites. In general, items where non-targets signal a low value of information (type 1) were supposed to be the easiest items, whereas items where the target link differs only marginally from non-target links in features signaling its relevance and credibility (type 2 to 4) should be more difficult (H1.1). Visiting a target’s website (i.e., target navigation) should facilitate solving the item correctly, as the target website is designed to provide information that marks the website as the best choice in terms of relevance and credibility (H1.2). On the contrary, there can be several reasons for visiting a non-target website (i.e., non-target navigation), from ensuring to not miss anything to just drawing inadequate inferences from the SERP information. We expected to see an overall negative effect of non-target navigation on the probability of task success, as it might indicate the result of inappropriate judgment (H1.3), but also a differential effect of non-target navigation in type 4 items (i.e., the incongruent condition where the website information fails the link information). As non-targets in these items were designed to look highly attractive, but disappoint when visited, non-target navigation should actually support students in discarding the attractive alternative (H1.4).

With regard to the nomological network of the EVON, we investigated the relations of students’ EVON performance with other variables. A test that claims to represent a skill to evaluate written information should mandatorily be associated with indicators of cognitive information processing. To examine this aspect, we investigated the relationship of the EVON with students’ graduation grades (German “Abiturnote”) as indicator of general cognitive performance and sentence-level comprehension as indicator of reading skill. German graduation grades are an aggregate of subject-specific grades assessed by several teachers over a couple of years. Accordingly, they do not reflect specific domain knowledge and are discussed as indicators of general cognitive abilities (e.g., Sorge et al., 2016). They also show a high predictive value for academic success (Trapmann et al., 2007). Note that lower numerical values of German grades indicate better performance. Reading skill is necessary to decode and understand written information. Unsurprisingly, reading skills on word, sentence, and text levels were shown to predict school students’ evaluation of online information (Hahnel et al., 2018). Therefore, we expected that the probability to solve an EVON item correctly increases by better (lower) graduation grades (H2.1) and higher reading skill (H2.2).

When investigating web search behavior, prior knowledge usually needs to be taken into account, as it supports web users in interpreting and evaluating semantic and message-related cues and contributes to both the assessment of relevance and credibility (e.g., Hölscher and Strube, 2000; Lucassen et al., 2013). Despite the importance of prior knowledge, however, we did not explicitly expect to find any effect of prior knowledge of the EVON topics on performance. Topic-specific knowledge might facilitate item processing, but due to the item design, it was not necessary to solve the items correctly. Nevertheless, we regarded prior knowledge as an important covariate.

### Method

#### Sample

A convenience sample of 173 students was recruited on the campus of a German university. Because of technical issues (e.g., server connection problems) or commitment (e.g., withdrawal from test), 21 cases were excluded, resulting in a final sample of 152 students (66.2% female) aged from 18 to 37 years (mean = 23.2, *SD* = 3.4). The participants were enrolled in different programs (54.7% bachelor, 14.0% master, 31.3% teacher training and others) from the humanities and social sciences, natural sciences, engineering sciences, economics, and medicine (semesters 1–19, mean = 6.9, *SD* = 3.7). Participants’ final school grades ranged from 1 (“very good”) to 4 (“sufficient”; mean = 2.3, *SD* = 0.7).

#### Procedure

The study was hosted on a server within our institute, on which the data of the participants were also collected and stored. Participants were recruited by posters on the campus, social media, and direct contact. Most students took an individual test session with a test administrator and received a small gift for participation (e.g., a candy or a ballpoint pen). To increase the reach of our recruitment, we also offered participants to conduct the test independently online; 15 students made use of this offer and received an invitation email with a link. Participation was voluntary and anonymous. After giving their informed consent, the participants were asked to complete a questionnaire assessing demographic variables and their educational background. Afterwards, the participants were asked to work on a speeded test assessing reading skill at sentence level as well as on the tutorial and the eight items of the EVON. Finally, the participants were requested to state how familiar they were with the topics of the EVON items. A test session took about half an hour.

#### Measures

##### Evaluating online information

Students’ performance on the EVON items was assessed in terms of dichotomous item scores (0 = incorrect, 1 = correct). The data showed 2.14% missing values in total (including omitted responses and not-reached items). Because of this small amount, missing values were treated as if the respective item had not been administered (Pohl et al., 2014). In addition to the item scores and based on students’ log files, it was assessed whether or not the students visited the target website (0 = no visit, 1 = at least one visit) or one or more of the non-target websites (0 = no visit, 1 = at least one visit). Across all cases (152 students × 8 items), the target was visited in 52.6% and the non-targets in 57.8% of cases.

##### Topic-specific knowledge

After the EVON assessment, the participants were asked to indicate how familiar they were with the topics in the EVON items. For each topic, they were requested to rate their previous knowledge and experience, responding on a 5-point Likert scale (1 = “don’t know what it is,” 2 = “heard of it,” 3 = “little prior knowledge,” 4 = “solid prior knowledge,” 5 = “excellent prior knowledge”). Across items, students reported little prior knowledge on average (mean = 3.13, *SD* = 0.49, min = 1.88, max = 4.38).

##### Reading skill

Reading skill was assessed by a sentence verification task that measures the ability to read accurately and quickly (i.e., automatized basic reading processes of lexical access and semantic and syntactic integration of propositions at sentence level; see Johnson et al., 2011; Zimmermann et al., 2014). The test consisted of 58 items that the participants were asked to evaluate as “true” or “false” as quickly and accurately as possible by pressing a respective button (α = 0.97; e.g., “Sugar is sweet,” “A cactus is a little furry animal”; Richter et al., 2012). The test has a total time limit of 80 s. The item contents draw upon common knowledge and are easy to understand (i.e., without uncommon words, complex syntactic structures, or specific knowledge requirements). The stimuli were half true and half false and varied in their semantic abstractness, the number of propositions (one to three propositions), and the sentence length (16–61 characters). The participants processed between 12 and all 58 sentences (mean = 41.1, *SD* = 11.9). The reading score was calculated as the number of correct responses minus the number of incorrect ones (mean = 39.9, *SD* = 12.1, min = 8, max = 58).

#### Data Analysis

For investigating the EVON assessment, a Rasch model was fitted on students’ item scores (Embretson and Reise, 2000). Relative frequencies of correct scores and descriptive point-biserial correlations of the item scores with the sum of scores were inspected. The fit of the Rasch model was examined by inspecting values of item infit and outfit (thresholds between 0.7 and 1.3; Wright and Linacre, 1994) and visual inspection of item characteristic curves and observed non-parametric response functions with respect to non-monotony and unexpected asymptotes. For testing the assumptions of local independence and unidimensionality, we examined Q3 statistics (cutoff: |value| > 0.2; Chen and Thissen, 1997) and conducted modified parallel analyses (Drasgow and Lissak, 1983).

For hypothesis testing, a series of generalized linear mixed models (GLMMs) was carried out (De Boeck et al., 2011). In these models, the probability of successfully solving an EVON item is predicted by fixed and random effects with regard to the hierarchical data structure of item responses nested in persons. Fixed effects are constant across observed units (e.g., students and items), while random effects vary across units. We specified a baseline model including a fixed intercept and random intercepts for students and items.

For examining the effects of item design and navigation behavior (H1.1–H1.4), the baseline model was extended to include fixed effects of the item types (model M1), of target navigation and non-target navigation (M2), and of both the item types and the navigation variables and an additional interaction of item types and non-target navigation (M3). The item type, target and non-target navigation were categorical variables with the reference categories of “type 1 (low attractiveness, congruent)” and “no navigation”.

For examining the nomological network of the EVON, the baseline model was extended by students’ graduation grades and reading skill (H2.1 and H2.2). Topic-specific prior knowledge was included as a person-by-item covariate. The continuous predictors were *z*-standardized before entered to the regression models. Accordingly, the regression coefficients represent the predicted change of the probability of task success when a predictor increases by one standard deviation in a logit metric.

The analyses were carried out in *R 3.5.3* (R Core Team, 2019) with the R packages *TAM* (Robitzsch et al., 2019; for IRT modeling) and *lme4* (Bates et al., 2015; for estimating GLMMs). The tests were one-tailed, with a type I error probability of 5%.

### Results and Discussion

#### Scaling

**FIGURE 2:**
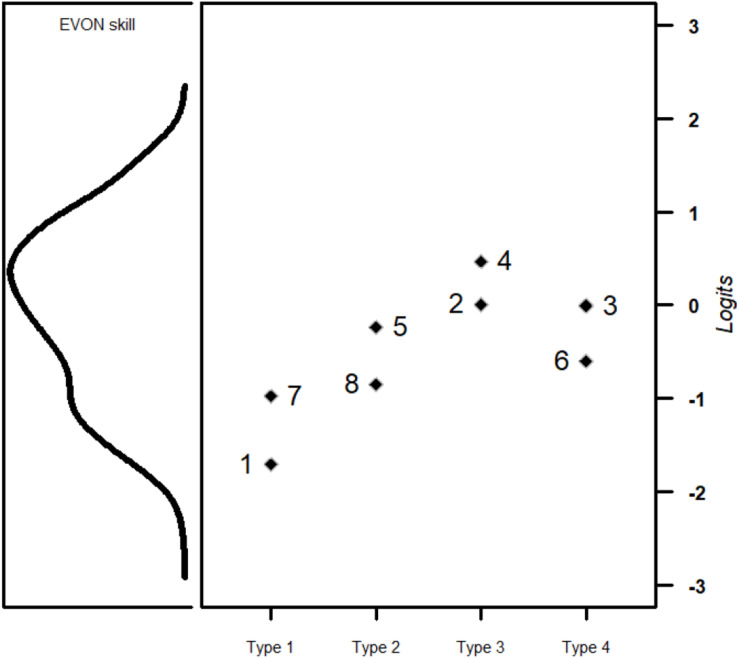
Wright map of the distribution of students’ skill in evaluating online information **(left)** mapped on the same scale as the difficulty of the EVON items **(right)**. Item difficulties are clustered according to the item type (*x*-axis; see Tables 1, 2).

Fitting a Rasch model, the estimated *expected a posteriori* (EAP) scores showed an EAP reliability of 0.62 (range of EAP scores = −1.99 to 1.46, variance = 1.14). Like in the pre-study, the items revealed relatively high rates of correct responses (Table 5). Figure 2 illustrates the estimated ability distribution of students simultaneously with the item difficulty parameters, underlining this lack of difficult items and indicating difficulty differences that seem to correspond with the item types.

**TABLE 5:** Results of item analyses.

**Item**	**% Correct**	***r*_pb_**	**Missing**	**Difficulty**	**Infit**	**Outfit**
1	62.2	0.54	4	–0.60	0.87	0.81
2	50.3	0.48	1	0.00	0.91	0.88
3	68.7	0.32	5	–0.97	1.02	1.05
4	40.9	0.35	3	0.47	1.03	1.04
5	80.5	0.21	3	–1.71	1.07	1.21
6	66.7	0.11	5	–0.85	1.18	1.34
7	55.0	0.44	3	–0.24	0.92	0.90
8	50.0	0.33	2	0.01	1.00	1.00

*r_*pb*_ is the point-biserial correlation of the item with the total sum score (excl. item).*

The visual inspection of item characteristic curves and the non-parametric response functions showed no severe model violations and even indicated an overfit for some items (i.e., a tendency to underestimate the probability of success for highly skilled students and to overestimate it for low-skilled students; see the Supplementary Material). Inspecting the infit and outfit values, item 6 revealed an outfit value beyond the threshold, indicating that it describes students of high or low skill poorly. Its point-biserial correlation with the sum score for all items was also rather low, but positive.

Supporting the assumption of local independence, the mean of all Q3 item pair statistics was slightly negative (−0.08). Only in four cases (14.3%), all involved item 6, a value above the cutoff was shown. The result of the modified parallel analysis was significant, indicating a violation of the unidimensionality assumption (second eigenvalue observed = 1.01, second eigenvalues averaged across 100 Monte Carlo samples = 0.75, *p* = 0.040). Without item 6, though, the result was opposite (second eigenvalue observed = 0.65, second eigenvalue sampled = 0.63, *p* = 0.401). Although the identified deviations of item 6 are not statistically negligible, they were still relatively small. Therefore and with respect to the construct representation, we decided to keep the item.

#### Analysis of the Item Type and Navigation Behavior

The GLMM baseline model showed an intercept of 0.48 (*SE* = 0.24), indicating that students’ probability to correctly solve an average EVON item was 61.7% (SD random person intercepts = 1.02; SD random item intercepts = 0.61). As also indicated in Figure 2 and in line with H1.1, the differentiation according to item types showed that students were most likely to correctly solve type 1 items and least likely to solve the other item types (Table 6, model M1). When the logit metric was transformed back into probabilities, the probability of correctly solving an average type 1 item was about 78.9%, which was reduced in items of type 2 (63.1%), type 3 (44.1%), and type 4 (57.3%).

**TABLE 6:** Results of the GLMMs examining the effect of item type and navigation on the probability of successfully solving an EVON item.

**Predictor**	**M1**	**M2**	**M3**
	**Est. (*SE*)**	**Est. (*SE*)**	**Est. (*SE*)**
Intercept	1.32(0.24)***	−0.55(0.28)*	0.37 (0.24)
Type 2	−0.78(0.30)**		−0.45(0.33)
Type 3	−1.55(0.30)***		−1.67(0.36)***
Type 4	−1.02(0.30)***		−1.62(0.35)***
Target navigation		2.69(0.03)***	2.86(0.25)***
Non-target navigation		−0.58(0.03)**	−0.20(0.33)
Non-target navigation × type 2			−1.49(0.44)***
Non-target navigation × type 3			−0.61(0.45)
Non-target navigation × type 4			0.21 (0.46)
SD random item intercepts	0.23	0.71	0.18
SD random person intercepts	1.02	0.43	0.44

*p < 0.05, **p < 0.01, ***p < 0.001.

With regard to navigation, the results of model M2 in Table 6 show that both target and non-target navigation significantly affected task success in an average EVON item, which is in line with H1.2 and H1.3. When students visited the target website, they were very likely to solve an average EVON item correctly (*b* = 2.69). In contrast, keeping the level of target navigation constant, non-target navigation was on average detrimental for students’ task success (*b* = −0.58). The tetrachoric correlation between target and non-target navigation was 0.86, indicating a general tendency to navigate or to not inspect the websites at all. The probability of task success without having navigated at all was 36.4% (intercept of M2), which is descriptively larger than the probability of guessing correctly on average in items with three or five response alternatives (26.7%).

Finally, the last model, M3 in Table 6, revealed—as predicted in H1.4—a differential positive effect of non-target navigation in item type 4 (*b* = 0.21), which, however, was not significant. The high standard error suggests that it might be a comparatively small effect that we cannot find as the item types are represented by only two items. Unexpectedly, there was a negative effect of non-target navigation in type 2 items, which means that the negative effect of non-target navigation was especially pronounced in these items.

#### Analysis of Relations to Other Variables

Before predicting students’ task success, we determined the correlations of the estimated EVON score with students’ graduation grades, reading skill, and the sum score of topic-specific knowledge ratings over all items. They showed that the EVON score significantly relates to better (lower) graduation grades [*r*(145) = −0.24, *p* = 0.004] and higher reading skill [*r*(150) = 0.25, *p* = 0.002]. Surprisingly, it was also negatively related to the overall sum score of students’ prior knowledge [*r*(147) = −0.24, *p* = 0.003], indicating that students who self-report a broad knowledge about all EVON topics would be less critical of search results.

The GLMM, investigating the effects of these variables on the probability of task success, explained a total of 12.90% of interindividual variation (SD random person intercepts = 0.96; SD random item intercepts = 0.63; intercept: *b* = 0.45, *SE* = 0.25, *p* = 0.068). In line with the hypotheses H2.1 and H2.2, students who were more likely to correctly solve an EVON item also showed significantly better (lower) graduation grades (*b* = −0.24, *SE* = 0.11, *p* = 0.033) and higher reading scores (*b* = 0.30, *SE* = 0.11, *p* = 0.007).

## Overall Discussion

With the aim of giving university students a first impression of their performance in evaluating online information, we developed a simulation-based achievement test for a MOOC that addresses the development of information literacy. In the present study, we reported on the development of the resulting instrument, the EVON. The test development and design of the interactive task environment followed a theory-based approach and distinguished four types of situations in which the use of certain heuristics is more or less suitable for making informed judgments about the appropriateness of information in search engine environments. Accordingly, the EVON claims to assess students’ skill to evaluate the relevance and credibility of such online information. In order to preliminarily validate this interpretation, we have analyzed several aspects concerning the response process, the internal structure of the instrument, and its relation to third variables.

With regard to the underlying response process, the pre-study showed that students identify and reflect on different aspects of the information provided based on semantic, structural, message-based, and sponsor-based cues. The resulting assessment of information relevance and credibility formed the basis for their selection of a link and its website. The results of the main study supported this assumption by showing different effects for different situations (item types). If supporting cues were identified early in the evaluation process and used appropriately, students were indeed able to make adequate predictive judgments beyond guessing based on the SERP information alone, as the average probability of task success without visiting a website suggests (36.4%). If the students’ decisions were enriched by evaluative assessments of website content, their chance of correctly solving the tasks increased, which is suggested by the positive effect of target navigation. In contrast, as indicated by the negative effect of non-target navigation, if their predictive judgments were inadequate, students may have turned their attention to less appropriate information and remained with it, perhaps because processing effort has already been made. This is also suggested by the unexpected but not implausible observation of the pronounced negative effect of non-target navigation in item type 2. If a website fails to meet web users’ expectations built up by predictive judgments, web users will find this source less trustworthy (Metzger and Flanagin, 2013). However, inadequate predictive judgments might be confirmed by the non-target website information in type 2, as it was not incongruent. The findings rather suggest that predictive judgments, once made, may already be quite robust. The positive effect of non-target navigation in item type 4 would have been in line with the empirical observation that web users rate websites as less trustworthy when their initial expectations are disappointed. However, as pointed out, it was not significant, potentially for reasons of the limited item set.

Insights into the internal test structure showed that the EVON sufficiently fitted a Rasch model, with the implication that it assesses a unidimensional construct. Although the results indicated minor difficulties with the psychometric properties of one item, as well as a lack of difficult items, these shortcomings can be overcome by adapting and refining the test on the solid foundation of the present test. To develop more difficult items, it might be worthwhile to create items that keep certain information features constant across links on the SERP (e.g., all website authors show the same level of expertise), thereby reducing the value that students can already gain from predictive judgments. For use in individual diagnostics, the development of further items is generally necessary, as this improves the reliability of the instrument and reduces the imprecision of the measurement. In summary, however, given the small number of items, the present psychometric results can be interpreted as acceptable for a standardized screening tool.

The investigation of evidence referring to the EVON’s relations to other construct-related variables showed weak but, as expected, significant relationships to cognitive performance measures such as graduation grades and reading skill. This indicates that the EVON reflects the cognitive performance of a person to some extent and adds to the empirical evidence on the relationship between reading and the evaluation of online information (Hahnel et al., 2018). Future research might extend investigations of the nomological network of the EVON score, especially with regard to motivational and personality-related aspects beyond cognitive variables. Studies on the use of digital media indicate that different online reading activities or specific motives underlying the use of digital media (e.g., information seeking vs. hedonic or social interaction purposes) are associated with mental processes of recognizing and interpreting web information (e.g., Lee and Wu, 2013; Senkbeil and Ihme, 2017; Senkbeil, 2018). Accordingly, it can be expected that the motivation of web users to process information has an impact on when and how they rely on certain heuristics affecting their credibility assessment of information (Metzger, 2007; Metzger and Flanagin, 2013).

Despite the overall promising findings supporting the test score interpretation of the EVON, the present attempt at validation can only be regarded as preliminary. Accordingly, there are a number of limitations that cannot be resolved by our study, but that also stimulate further research based on our findings. First, further validity evidence needs to be investigated, for example, on students’ EVON performance together with other measures of their information literacy or evaluation skill. Demonstrating positive relationships between the EVON and such skills would provide other strong validity arguments. A promising candidate for providing detailed insight into processes assessed by the EVON are, for instance, facets of source evaluation, such as the identification of source features, the evaluation of author credentials and the actual use of source information (e.g., Potocki et al., 2020). Positive relationships should emerge between students’ EVON performance and these facets of source evaluation skills, as the EVON claims to assess students’ assessment of credibility based on the identification and critical evaluation of source information. In this regard, it is noteworthy that the EVON might not reflect “typical behavior” of students dealing with online information (see Klehe and Anderson, 2007). As students should perform at their best (power test), we explicitly requested them to select a useful and trustworthy link. Without such an instruction, students might have paid less attention to information credibility. Although our results do not speak against interpreting the EVON score in terms of “typical behavior,” our validation arguments are weak in this respect. To validate such an interpretation, for example, an experiment would be needed in which one group works on the current EVON test and another group on the EVON test without the instruction amendment on trustworthiness.

Second, we scored the students’ answers dichotomously, but this does not mean that a more nuanced coding would not be possible. In particular, we see two directions for improvement, which could also be combined. On the one hand, enriched information could be obtained from alternative response formats, for example, by asking students directly about their perception of why a website appears to be more or less credible or by asking them to rate the relevance and credibility of each link. This option could easily be added to the EVON (e.g., in the form of a separate test part). On the other hand, the stimulus material could be further developed to the extent that it allows partially correct or even multiple correct response options. A partial credit coding might acknowledge responses that demonstrate moderate assessment skills but still show a lack of thoroughness, rigor, or critical thinking. The challenge would then be to construct such websites that would distinguish between moderate and high levels of competence. Given our psychometric results, which show a lack of difficult items, and recent proposals to consider aspects of critical thinking research (van Zyl et al., 2020), the checklist approach may have limited potential to meet this challenge. However, a more promising attempt might be to develop items that require students to identify and evaluate knowledge claims of websites and evidence that speaks for or against these claims. With respect to both directions, our article shows that EVON provides a solid basis for pursuing such developments.

Third, we only investigated the German version of the EVON. The test is available in five other languages. Although the other language versions do not automatically restrict the applicability of our findings, they should be subject to empirical testing for establishing measurement invariance between the different versions. Measurement invariance ensures that a test measures the same latent construct across several groups. Accordingly, it is an important prerequisite for comparability. Therefore and with respect to restrictions due to our small-scale convenience sample, further research is needed to investigate the generalizability of our findings.

Finally, the EVON was conceptualized as a screening instrument. Accordingly, the ILO MOOC currently uses the EVON as a warm-up test for a lesson on the subject of information evaluation, without further consequences for the course. However, there are possible other uses for which the EVON might be suitable after further adaptation. The EVON might be extended and adapted to serve as a preintervention–postintervention measure to investigate the effectiveness of interventions, such as technology-assisted trainings of evaluating information (for overviews, see Bråten et al., 2018; Braasch and Graesser, 2020). Based on the comprehensive item content and the process data collected during an EVON assessment, it might be even worthwhile to implement a feedback component that provides students not only with their EVON test score or raw item responses, but also information on why a selected alternative might have been suboptimal or how students approached the EVON tasks for purposes of self-reflection. For sure, the usefulness of such feedback for learners would need to be investigated. Yet, if it is found to improve students’ evaluation skill, the EVON has the potential to provide elaborate feedback to learners for improving a critical aspect of their information literacy.

In summary, with the EVON, we constructed a complex interactive assessment with an authentic task environment. We observed supporting evidence that its items elicited students to make use of different information features and employed various heuristics for assessing the relevance and credibility of information. Although our findings also uncovered a few weaknesses, and the efforts of validating the interpretation of EVON outcomes still need to be continued, the overall results speak in favor of a successful test construction and provide first indications that the EVON assesses students’ skill in evaluating online information in search engine environments.

## Data Availability Statement

The raw data supporting the conclusions of this article will be made available by the authors, without undue reservation, to any qualified researcher.

## Ethics Statement

Ethical review and approval was not required for the study on human participants in accordance with the local legislation and institutional requirements. The patients/participants provided their written informed consent to participate in this study.

## Author Contributions

CH: concept, coordination and item development, study design, data collection and preparation, analysis, and writing. BE: item development, data collection and preparation, analysis, and writing. FG: preparation of the manuscript. All authors contributed to the article and approved the submitted version.

## Conflict of Interest

The authors declare that the research was conducted in the absence of any commercial or financial relationships that could be construed as a potential conflict of interest.

## Footnotes

1 https://informationliteracy.eu/en

2 The CBA ItemBuilder is an authoring tool to create dynamic and interactive assessment and learning environments. It is free of charge and can be requested from the Centre for Technology-Based Assessment at DIPF (ib-support@dipf.de). https://tba.dipf.de/en/infrastructure/software-development/cba-itembuilder/cba-itembuilder-1?set_language=en

3 The names of all participants are fictitious.
