# Supplementary material for: Evaluation of Online Information in University Students: Development and Scaling of the Screening Instrument EVON
Source: Front Psychol. 2020 Dec 16;11:562128. doi: 10.3389/fpsyg.2020.562128 (PMC7773327; doi:10.3389/fpsyg.2020.562128)
Supplement: Supplementary file 1 [file Data_Sheet_1.PDF]

Supplementary Material: Item Characteristic Curves and Observed Response Functions

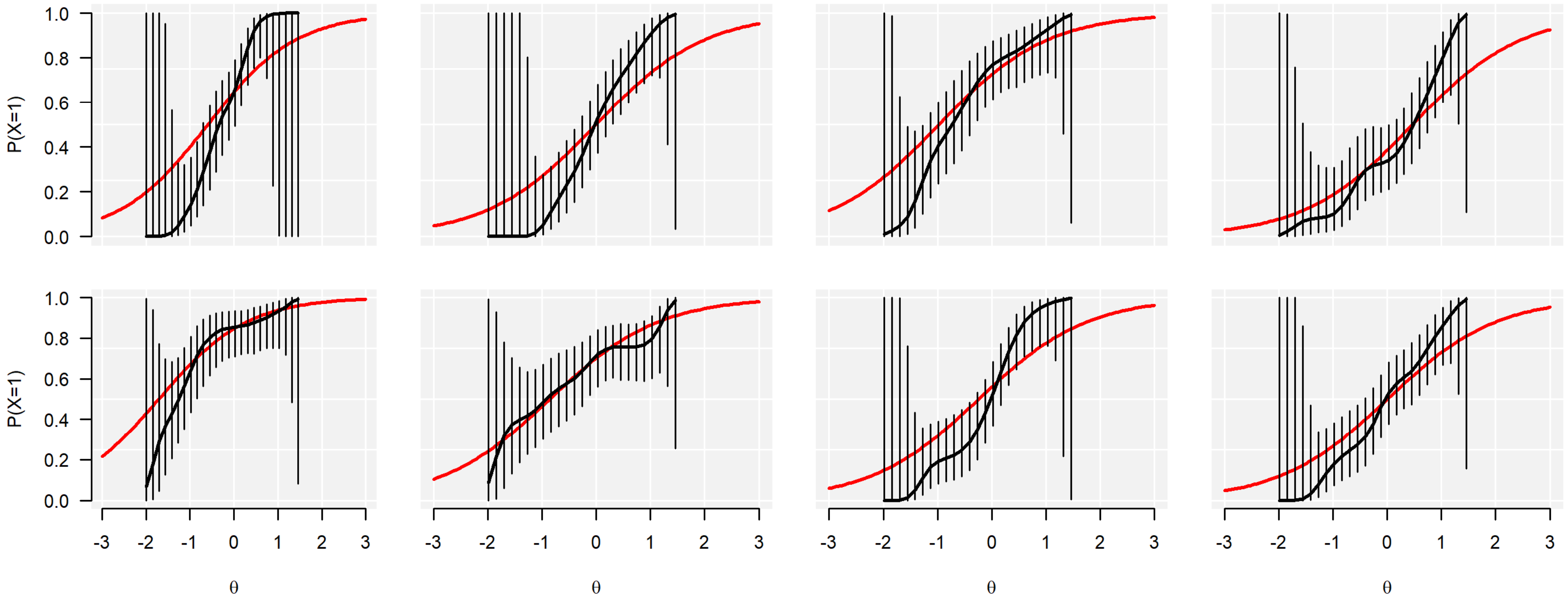

Supplementary Figure 1. Item characteristic curves (red) and observed non-parametric response functions (black) of the EVON items. From left to right: Item 1 to 4 in the top row; item 5 to 8 in the bottom row. The vertical confidence bands reflect the uncertainty of estimation. The functions were created with the R package *irtoys* (Partchev & Maris, 2017).

References

Partchev, I., & Maris, G. (2017). *irtoys: A Collection of Functions Related to Item Response Theory (IRT)*. https://CRAN.R-project.org/package=irtoys
